# Dickkopf1 - A New Player in Modelling the Wnt Pathway

**DOI:** 10.1371/journal.pone.0025550

**Published:** 2011-10-12

**Authors:** Lykke Pedersen, Mogens Høgh Jensen, Sandeep Krishna

**Affiliations:** 1 Center for Models of Life, Niels Bohr Institute, Copenhagen, Denmark; 2 Theory and Modelling of Biological Systems, National Centre for Biological Sciences, Bangalore, Karnataka, India; Centre for Genomic Regulation (CRG), Universitat Pompeu Fabra, Spain

## Abstract

The Wnt signaling pathway transducing the stabilization of *β*-catenin is essential for metazoan embryo development and is misregulated in many diseases such as cancers. In recent years models have been proposed for the Wnt signaling pathway during the segmentation process in developing embryos. Many of these include negative feedback loops where Axin2 plays a key role. However, Axin2 null mice show no segmentation phenotype. We therefore propose a new model where the negative feedback involves Dkk1 rather than Axin2. We show that this model can exhibit the same type of oscillations as the previous models with Axin2 and as observed in experiments. We show that a spatial Wnt gradient can consistently convert this temporal periodicity into the spatial periodicity of somites, provided the oscillations in new cells arising in the presomitic mesoderm are synchronized with the oscillations of older cells. We further investigate the hypothesis that a change in the Wnt level in the tail bud during the later stages of somitogenesis can lengthen the time period of the oscillations and hence the size and separation of the later somites.

## Introduction

A segmented body plan is a fundamental characteristic feature of vertebrates. The process of segmentation is carried out by a combination of changes in gene expression and relative anterior-posterior cell position in the presomitic mesoderm (PSM) [1]. In the anterior end of the embryo the somites are segmented at equally separated time points with species dependent periods. In mice the period is around 120 min and in frogs it is around 90 min.

In 1976 Cooke and Zeeman [2] proposed the clock and wavefront model to describe the segmentation process. The idea is that locally coupled oscillators are controlled by a morphogen gradient in the PSM. The oscillators are the clocks providing temporal information, e.g., cycle state, and the morphogen gradient is the wavefront providing spatial information about axial position. Until now three major pathways controlling the segmentation process have been found: the Notch, Wnt and FGF pathways. They all have target genes, which oscillates and, interestingly, Wnt target genes oscillate out of phase with Notch and FGF target genes [3]. These three pathways could be the clocks. There are decreasing gradients of wnt3a and fibroblast growth factor 8 (fgf8) starting from the tail bud through the PSM [4], [5]. The two gradients act in synergy with each other during the somitogenesis [6], [7]. The actual setting of the somites happens at the determination front, where the fgf8 level reaches a certain threshold. Cells past this determination front become permissive to form somites depending on their phase of oscillation [8].

In 2003 it was discovered by Aulehla et al. [4] that Axin2 oscillates during the segmentation process in developing mouse embryos. Since their discovery several models for the Wnt oscillator have been proposed [9]–[11] with Axin2 as a key variable. However, while Axin2 is a negative regulator of the Wnt pathway, mice with a null mutation of Axin2 do not exhibit a segmentation phenotype – only malformations of skull structures [12]. Therefore, we propose a new model for the core negative feedback loop generating oscillations in the Wnt pathway, with Dickkopf1 (Dkk1), rather than Axin2, closing the feedback loop. Dkk1 has an oscillatory behavior during the segmentation process in mouse embryos [3] and lowered expression of Dkk1 results in smaller and more irregular vertebrae in mice [13], [14]; similar to the phenotype produced by overexpression of Wnt3a.

## Analysis

### Modeling the Wnt/*β*-catenin pathway

During Wnt signaling *β*-catenin interacts with the TCF/LEF-1 DNA-binding proteins to promote transcription of Wnt target genes [15], [16]. As for Axin2 the transcription factor for Dkk1 is *β*-catenin [17], [18]. After transcription and translation Dkk1 goes through the cellular membrane where it can bind to the extracellular domains of the low-density lipoprotein receptor-related protein 5 and 6 (LRP5/6). When bound to LRP5/6, Dkk1 acts as an inhibitor of Wnt signaling by blocking the association between Wnt, Frizzled (Fz) and LRP5/6 [19]. Wnt acts as an inducer for the formation of this complex and Dkk1 is a competitor to this induction [20], [21].

It has been proposed that the Wnt signal is transduced through the cell membrane by the binding of Dishevelled (Dsh) to the intracellular domain of the Fz receptor [22]. Axin and Dsh can bind together via their DIX domains [22] and they co-localize at the membrane [23] during Wnt signaling. Therefore Dsh bound to Fz may recruit Axin bound to the glycogen synthase kinase 3 (GSK3*β*) to the LRP5/6 receptor [24], where a phosphorylation of LRP5/6 is initiated. The LRP5/6 receptor has a binding site for Axin and upon Wnt signalling GSK3*β* (bound to Axin) phosphorylates LRP5/6, which requires Axin [24]. The phosphorylated LRP5/6 receptor may be able to recruit and more efficiently bind the Axin-GSK3*β* complex to the membrane and the phosphorylation process is thereby amplified [25].

At the cell membrane Axin is phosphorylated by GSK3*β* and then degraded [26], [27]. The degradation of Axin leads to a decrease in the formation of the destruction complex comprised of *β*-catenin, the two kinases GSK3*β* and casein kinase (CKI*α*), and the scaffolding proteins Axin and adenomatous polyposis coli (APC). In the destruction complex *β*-catenin gets phosphorylated and subsequently degraded.

Interestingly enough GSK3*β* plays a dual role in controlling the Wnt signal. When the Wnt signal is off then GSK3*β* phosphorylates *β*-catenin in the destruction complex and when the signal is on then it phosphorylates Axin at the LRP5/6 receptor. Whether it is the same or distinct Axin-GSK3*β* complexes that carry out the phosphorylation of *β*-catenin and Axin is unknown [28].

**Figure 1:**
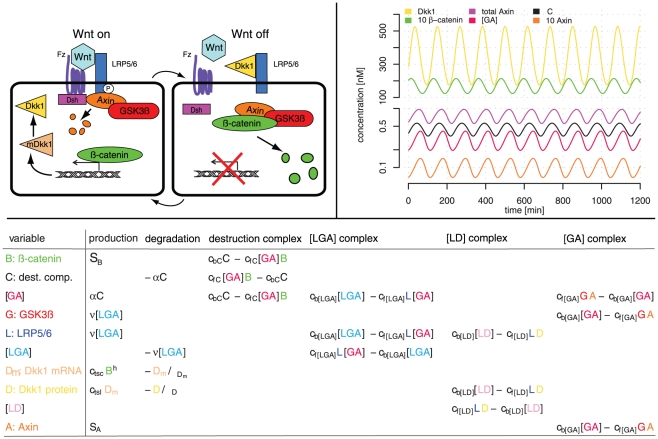
The diagram, simulation and equations of the Wnt model. (Top,left): A diagram of the Wnt model with a feed-back loop over Dkk1. Included are only members of the Wnt pathway that are important for the understanding of the negative feedback loop. When the Wnt signal is on, then Axin gets degraded at the LRP5/6 complex and *β*-catenin can act as a transcription factor of the Wnt inhibitor Dkk1. Vice-versa, when the Wnt signal is off, due to inhibition by Dkk1, then *β*-catenin gets degraded. (Top,right): Simulated time series for a selection of variables from the model listed in the bottom panel. The total level of Axin (magenta) is low, which complies with the findings of [10]. (Bottom): Equations of the Wnt model split up in terms describing production, degradation and complex dynamics.

Our model does not include the dynamics of the kinase CK1*α*, the scaffolding protein APC, the DNA binding proteins TCF/LEF-1 and the protein Dsh, since their dynamics are not a major part of the negative feedback loop. The dynamics of CK1*α* and APC are included in the parameters governing the destruction complex, the TCF/LEF-1 dynamics are contained within the transcription of Dkk1, and the dynamics of Dsh are included in the formation of the complex consisting of Axin, GSK3*β* and LRP5/6 at the cell membrane. Figure 1 shows a simplified diagram of the proposed model and the associated equations. The variables *C*, [*GA*], *G*, *B*, *L*, *D*_m_, *D*, [*LD*], *A* and [LGA] are the concentrations of the destruction complex, GSK3*β*-Axin complex, GSK3*β*, *β*-catenin , LRP5/6, Dkk1 mRNA, Dkk1 protein, Dkk1-LRP5/6 complex, Axin and LRP5/6-Axin-GSK3*β* complex. The formation and breaking of a complex *X* are denoted by 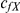 and 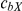, respectively. The transcription and translation rates of Dkk1 are given by the parameters 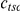 and 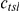, respectively. The Hill coefficient on *B* regarding the transcription of *D_m_* is associated to the amount of cooperativity between *β*-catenin and the TCF/LEF-1 complex. For example, no cooperativity would result in a Hill coefficient of one.

The concentration of GSK3*β* has been shown to be extremely stable [10] and consequently its total concentration, 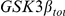, is assumed to be constant during the time scales considered. The same assumption goes for the total concentration of LRP5/6, since the half-life of LRP6 is around 4.7 hours [29]. Therefore we have not included any source or sink for the concentrations of *G* and *L*. Only constitutive sources, *S_B_* and *S_A_*, of *β*-catenin and Axin, respectively, are included in the model, because free (unphosphorylated) *β*-catenin and Axin is stable [10].

### Determination of the parameter values

The parameters used for our model are listed in Table 1. In Ref. [10] a model for the Wnt pathway in *Xenopus* is proposed and from this article estimates for the dissociation constants for the destruction complex (*C*) and the [*GA*] complex, the degradation of *β*-catenin (*α*), the total concentration of GSK3*β*, and the sources of Axin and *β*-catenin are taken. Dkk1 binds to LRP5/6 with a high affinity; the dissociation constant has been measured to be around 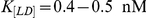 [19], [20].

**Table 1:** Parameters in our model of the Wnt system and their default values.

Parameter	Process	Default Value
*K_C_*	Dissociation constant *C*	8 nM
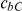	Breaking of *C*	7 min^−1^
*α*	Degradation of *β*-catenin	2.2 min^−1^
*K*_[*GA*]_	Dissociation constant [*GA*]	1.5 nM
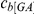	Breaking of [*GA*]	4 min^−1^
*K*_[*LGA*]_	Dissociation constant [*LGA*]	1 nM
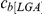	Breaking of [*LGA*]	10 min^−1^
*v*	Degradation of Axin	3.8 min^−1^
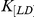	Dissociation constant [*LD*]	0.5 nM
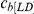	Breaking of [*LD*]	0.02 min^−1^
*S_B_*	Constant source of *β*-catenin	1 nM/min
*S_A_*	Constant source of Axin	0.02 nM/min
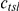	Transcription of *dkk1*	0.02 min^−1^
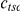	Translation of Dkk1 mRNA	0.025 (Mm^2^ min)^−1^
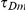	Average lifetime of dkk1 mRNA	8 min
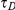	Average lifetime of Dkk1	16 min
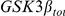	Total G level	45 nM
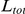	Total L level	15 nM

The other parameters are estimated to produce oscillations with a period of around 120 min and a very low concentration of total Axin as found by Ref. [10]. The low concentration of total Axin is thought to act as a buffer to changes in the concentration of the other constituents, which may also take part in other signaling pathways [30].

In the activation of transcription of Dkk1 by *β*-catenin we assume cooperativity between *β*-catenin and TCF/LEF. The Hill coefficient is set to three. A Hill coefficient of two can produce oscillations, but this requires the affinity of *β*-catenin to the [GA] complex to be much higher than suggested by the dissociation constant value used in Ref. [10]. Therefore, we preferred to retain the parameter values of Ref. [10] but increase the Hill coefficient to three. In other models of the Wnt pathway, Hill coefficients of values two and five have been used [9], [10], [31].

## Results and Discussion

### Oscillations of the mRNA, protein and complex levels

The parameter values produce oscillations of the involved constituents with a period of around 120 min as seen in Fig. 1. In addition, the concentration of Axin is very low compared to the concentrations of the other variables, as discussed above. The phase shifts between the different oscillatory components of the Wnt model can be explained by considering the sequential steps of the model. An increase in the [LGA] concentration leads to a decrease in the Axin concentration. This decrease causes a reduction in the concentration of the destruction complex and consequently an increase in the *β*-catenin concentration. This increase will after a while cause an increase in the Dkk1 concentration, which leads to an increase in the [*LD*] concentration. The high affinity of the [*LD*] complex leaves little free LPR5/6 behind to form a complex with [GA]. Thus the concentration of the [LGA] complex decreases, leading to an increase in Axin, and the cycle continues. The concentrations of GSK3*β* and [*GA*] are mirrors of each other, since a high concentration of [*GA*] will leave less free GSK3*β* behind.

Ref. [32] found no significant oscillations in the level of *β*-catenin . For our choice of parameters *β*-catenin shows an oscillatory behavior with an amplitude of approximately 5nM, which is not significantly low. A different set of parameters could possibly give a smaller amplitude of *β*-catenin but the general results, presented later, are not significantly altered by this. Even though *β*-catenin does not oscillate it has been shown that the Notch target gene Nrarp, which stabilizes LEF-1 [33], does oscillate [3]. LEF-1 does not oscillate in the PSM [34]. Notch and Wnt target genes oscillates out of phase. Thus, when Dkk1 is high, resulting in the inhibition of Wnt signaling, Nrarp will have a low expression, resulting in LEF-1 ubiquitination and consequently less Dkk1. The oscillatory behavior seen in Fig. 1 nearly resembles this. Thus, the *β*-catenin variable in our model can be considered as a coarse-grained variable combining the effects of *β*-catenin , Nrarp and LEF-1.

### Stability of the period and amplitudes of oscillations

**Figure 2:**
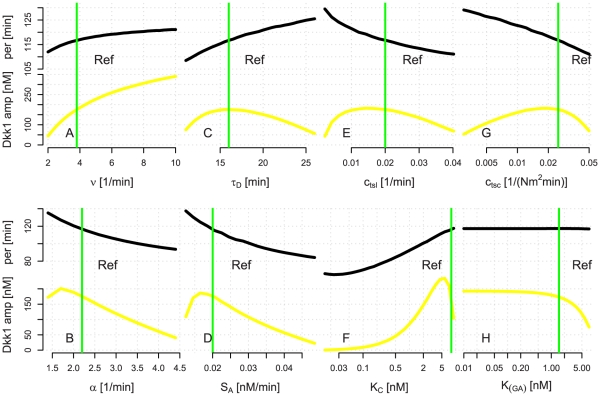
Changes in the periods and amplitudes when varying parameter values. The period (black) and amplitude (yellow) of Dkk1 oscillations as *v* (A), *α* (B), 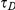 (C), *S_A_* (D), 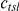 (E), 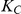 (F), 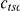 (G) and 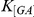 (H) are varied. The green lines refer to the values listed in Table 1.

In Fig. 2 the period and amplitude of the Dkk1 oscillations are plotted as a function of a selection of parameters. Changing *K*_C_ results in the most drastic changes in amplitude and period of Dkk1 (Fig. 2F). Even though the assembling of GSK3*β* and Axin is involved in three out of the four complexes in our model, changes in *K*_[*GA*]_ do not affect the period and only for *K*_[*GA*]_>1 is the amplitude of Dkk1 oscillations really affected, see Fig. 2H.

The degradation of both Axin and *β*-catenin affects the amplitude. If more *β*-catenin is degraded then less Dkk1 is transcribed (Fig. 2B). Vice versa with the degradation of Axin. If more Axin gets degraded then less *β*-catenin gets phosphorylated at the destruction complex resulting in more transcription of Dkk1 (Fig. 2A). As these parameters affect the system in opposite directions, an experiment where the capability of GSK3*β* to phosphorylate Axin and *β*-catenin is tested, could shed light on the dual mechanism of GSK3*β* in the Wnt pathway.

The half-life of Dkk1 affects the period – a shorter half-life leads to a shorter period (Fig. 2C). A similar effect of Axin2 half-life was seen in a previous model with an Axin2 negative feedback loop [11] similar to our Wnt model with a negative Dkk1 loop. Shorter periods are also found when the translation and transcription rates are increased (Fig. 2E,G). In comparing Figs. 2B,D it can be seen that altering *α* is almost the same as altering *S_A_*, which makes sense since the concentration of *C* is dependent on both the source of Axin and the rate of *β*-catenin phosphorylation.

The amplitude of Dkk1 oscillations differs between the case with *h* = 3 and *h* = 2, compare Figs. 1 and S1. Figure S1 shows the oscillations for *h* = 2 and the parameters used for *h* = 2 are listed in Table S1. A measure of the actual size of the Dkk1 amplitude along with a measure of the kinetics of the destruction complex could give a hint of the cooperativity between *β*-catenin and the TCF/LEF-1 complex. If the real Hill coefficient is two, then our model predicts that to produce oscillations they must also bind with a high affinity, and that the resultant oscillations of Dkk1 will have a relatively smaller amplitude than if the Hill coefficient is three.

### A spatial Wnt gradient induced by time variation

The oscillations of the variables in our model could function as the segmentation clock postulated in the clock and wavefront model [2]. We now investigate whether such oscillations can consistently be coupled to a wavefront, i.e. a spatial gradient of Wnt. As oscillating cells move through the PSM they effectively see a decreasing level of Wnt in time. Therefore we model the spatial Wnt gradient simply by a time dependent decrease in the parameter 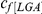. Other models have also been proposed with a gradient of a morphogen protein [35]-[37]. These models are complementary to ours in that they use abstract models of the clock, not any concrete mRNA, proteins and complexes interacting as in our model.

The reason for using 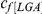 as the time dependent parameter to mimic a Wnt gradient is clear if we introduce the variable [*LW*] describing the binding of Wnt (*W*) to the LRP5/6 receptor:
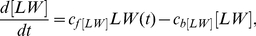
(1)


and if we assume steady state for the binding of Wnt to LRP5/6 then
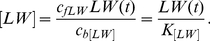
(2)


When substituting [*LW*] for *L* in the equations with terms governing the formation of the [*LGA*] complex, it can be seen that the rate constant for formation of [*LGA*] will be time dependent
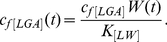
(3)


**Figure 3:**
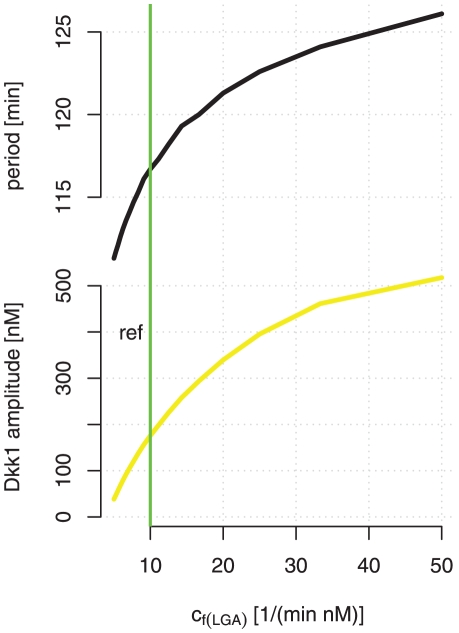
The effect of the Wnt level on the period and amplitude. The amplitude (solid) and the period (dashed) of Dkk1 oscillations for varying values of the parameter 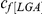. The green vertical line denotes the value of 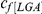 in the reference state.

For simplicity we will assume that the Wnt gradient, and hence 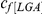, has a Gaussian profile, which is what one would expect if the gradient was determined mainly by diffusive processes. For reference, Fig. 3 shows the amplitude and period of Dkk1 concentration for a range of 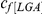 values. The green line refers to the reference value of 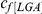; reducing it results in smaller amplitudes.

The length of the PSM is approximately constant during the formation of the first 15–20 somites in mice embryos [38]. The same is almost true for the size of the somites. At this stage the somites are ∼100 *µ*m and the PSM is about 1 mm, i.e. the PSM has a length corresponding to the length of 10 somites. Thus, a cell budded off in the tail bud at this stage will be segmented in around ∼1100–1300 min.

It has been measured that FGF (regulated by Wnt) exhibits a gradient in the PSM with a fold change of two to five [5]. The fold change used for 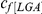 is two. Assuming that Wnt diffuses through the PSM and setting the final value of 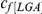 equal to 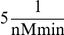 enable us to calculate a Gaussian profile of 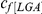 representing the Wnt gradient. In Fig. S2, Gaussian profiles of 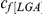 in the PSM are plotted with different initial values. A decreasing value of 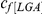 in the PSM will give rise to smaller amplitudes and slightly shorter periods. Experimentally it has been shown that the wavelength of the oscillations in the PSM decreases from the tail bud to the determination front [39]. If the oscillations are proportional to the wavelength, then the steepness of the 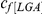 profile sets the pace with which the wavelength decreases.

### Synchronization of neighbouring cells

**Figure 4:**
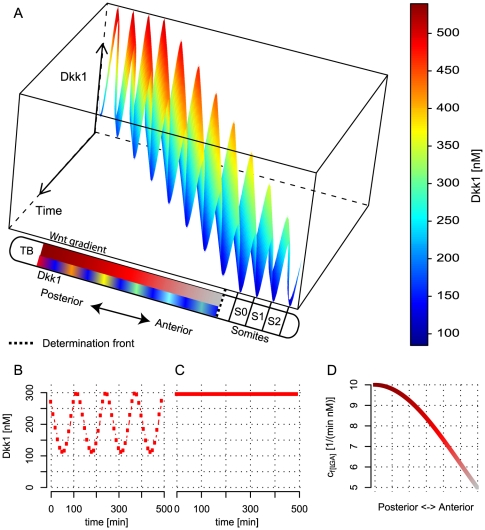
Synchronization of neighboring cells. (A) Time series for the Dkk1 concentration. Space is introduced by letting a cell bud off from the tail bud every 10th min. Thus the cells move relatively in the PSM. At the determination front the oscillations arrest. (B/C)The level of Dkk1 at the determination front with *R* = 10 min and synchronization between neighboring cells and with *R* = 10 min and the cells have the same initial level of Dkk1 (C). (D) The Gaussian profile of 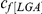 in the anteroposterior direction. TB: tail bud. S*_i_*: Somite *i*, where S_0_ is the newly formed somite.

Figure 4A shows the Dkk1 oscillations in an elongating embryo with a decreasing 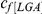 as described above. The elongation of the embryo is modeled in a very simplified manner. It is considered in only one dimension and a cell buds off from the tail bud at regular time intervals denoted by *R*. In Fig. 4 we use *R* = 10 min. We further assume that the initial state of a newly budded cell in the PSM is the same as that of its anterior neighboring cell, i.e. if the state for a cell *i* at time *t* is denoted 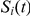, then the initial state of a cell *i*+1 is 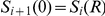. Thus, we effectively introduce a synchronization in the oscillations of adjacent cells. But this is put in by hand, rather than by an explicit coupling between cells in the model. The final level of Dkk1 at the determination front is oscillating (see Fig. 4B with a period of 120 min. It is the built-in synchronization that is the reason behind this. If, instead, all the cells were assigned the same initial state, i.e. 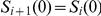, then the levels of Dkk1 at the determination front would also be equal, as in Fig. 4C. In a real embryo the synchronization, of course, occurs through coupling of the individual clocks in the PSM cells [40], which we have not modeled. However, the way we put in synchronization in our model is sufficient to demonstrate that its presence is necessary for a proper function of the segmentation process. The importance of synchronization has also been shown experimentally [41].

One could imagine that the synchronization of the clocks is not perfect. If the initial state is randomly chosen within the whole range of Dkk1 levels, then the oscillations of Dkk1 at the determination front are disrupted and no periodicity is visible (see Fig. S3B). If the initial state of a cell *i*+1 is chosen randomly within the interval 
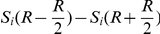
, then the period is almost unaltered (see Fig. S3A). Thus, the system seems to be robust to small changes in the synchronization.

In zebrafish the mechanism of synchronization is well understood by Delta-Notch interactions [42]. In the literature we have found models which couple the cells in zebrafish [40], [42]–[45] by various mechanisms, such as coupling of phase oscillators and coupling of oscillating clock genes with a signalling protein. The model of Ref. [44] shows that even a weak coupling helps synchronization. In the embryo, mitosis and stochastic gene expression could result in nonlinear noise [40] that could disrupt the synchronization if they are not coupled strongly enough.

In the above simulations, a Gaussian profile of 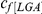 is used, since diffusion of Wnt is thought to be the main reason for the Wnt gradient in the PSM. The profile of 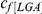 would still be Gaussian if we also included a half-life of Wnt. If the Wnt gradient was only controlled by the half-life of Wnt then the levels of Dkk1 still oscillate at the determination front with a period of 120 min, when the model is simulated as above with *R* = 10 min.

Because Ref. [32] found a decreasing gradient of *β*-catenin in the PSM, we also tried giving *S_B_* a Gaussian profile in the PSM with a fold change of two from the tail bud to the anterior part of the PSM. This did not alter the 120 min oscillations of Dkk1 at the determination front, when the model was simulated as above with *R* = 10 min.

### A decreasing Wnt level in the tail bud and an increase in period of segmentation

It is known that the period of somite formation increases [38] during late stages of somitogenesis in various organisms. In 2004 Aulehla and Hermann [46] hypothesized that an increase in the Wnt level of the tail bud could result in longer periods of the segmentation process observed in mice embryos. In our model, simulating an increasing level of Wnt (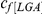 from 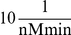 to 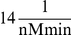) in the tail bud we do see that the period initially lengthens and the amplitude increases, see Fig. S4B. However, if the Wnt level is increased further the period decreases to as low as 30 min. An experiment where Wnt is upregulated in the tail bud would elucidate their hypothesis and our findings.

Recent experiments with chick embryos show the opposite – Wnt is downregulated in the tail bud at late stages of somitogenesis [34]. In our model, simulating a linear decrease of Wnt, through the parameter 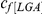, from 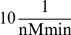 to 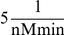, causes the period of Dkk1 oscillations to increase. The mechanism behind this increase in the period is different from that causing a small increase in the period as described above. There the Wnt level was decreased throughout the PSM, where here the initial level of Wnt in the tail bud is decreased, i.e. the gradient of Wnt in the PSM becomes less steep. The amplitude of the Dkk1 oscillations decreases at the same time, which is expected because the segmentation process does stop as the Wnt level diminishes.

### Outlook

We propose a Wnt model with Dkk1 as the core for the negative feedback loop which exhibits sustained oscillations of Dkk1. The clock and wavefront model was investigated using the Dkk1 oscillations as the clock and a Wnt gradient in the PSM as the wavefront. By simulating the elongating embryo we were able to test the importance of synchronization between neighboring cells. In addition, we could also show that small errors in the synchronization did not significantly disrupt smooth oscillations of the Dkk1 levels at the determination front. We could reproduce the experimental finding in chick embryos that downregulation of Wnt in the tail bud might lengthen the oscillation time periods during late stages of somitogenesis. The negative feedback loop involving Dkk1 introduced produces very similar behavior as the Axin2 negative feedback loops modeled previously. Thus, it is conceivable that these two loops function together, providing some redundancy with respect to each other. This could explain why Axin2 null mutant mice do not show any segmentation phenotype and Dkk1 null mutant mice show only some irregularity in the vertebrae. Such redundancy has been seen in the case of fgf4 and fgf8. Neither are individually essential, but removing both disrupts somitogenesis [7]. It would there be interesting to see what phenotype a double knockout of both Axin2 and Dkk1 exhibits.

## Supporting Information

Figure S1 **Oscillations of the Wnt model with h = 2**. The Wnt model is simulated with a Hill coefficient of *h* = 2. The model still shows oscillations with a period of around 120 min, but the the affinity of the *β*-catenin to bind the [*GA*] complex needs to be much higher than expected from experiments. The parameters used in this simulation ca be found in Table S1.
(PDF)

Figure S2
**Gaussian profiles of the Wnt gradient**. (Top): Gaussian profiles of 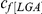 in the PSM are plotted with different initial values. A decreasing value of 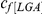 in the PSM will give rise to smaller amplitudes (middle) and slightly shorter/ almost constant periods (bottom). The abbreviation ref±*i* denotes the reference state value of 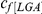 ±*i*.(PDF)

Figure S3 **Desynchronization of neighboring cells**. If the desynchronization between neighboring cells is small (A) then the oscillations of the Dkk1 level at the determination front is almost unaltered. However, if the desynchronization is strong then these oscillations are not appearing.
(PDF)

Figure S4 **The Wnt level in the tail bud**. (A): When the Wnt level decreases in the tail bud, then the period of the Dkk1 level at the determination front extends. (B): The period is also extended when the Wnt level increases in the tail bud, but the periods drop significantly below the period in the reference state.
(PDF)

Table S1 (PDF)
