# Supplementary material for: Dickkopf1 - A New Player in Modelling the Wnt Pathway
Source: PLoS One. 2011 Oct 12;6(10):e25550. doi: 10.1371/journal.pone.0025550 (PMC3192063; doi:10.1371/journal.pone.0025550)
Supplement: Figure S1 — Oscillations of the Wnt model with h = 2. The Wnt model is simulated with a Hill coefficient of h = 2. The model still shows oscillations with a period of around 120 min, but the the affinity of the β-catenin to bind the [GA] complex needs to be much higher than expected from experiments. The parameters used in this simulation ca be found in Table S1. (PDF) [file pone.0025550.s001.pdf]

Figure S1 - Oscillations of the Wnt model with h=2

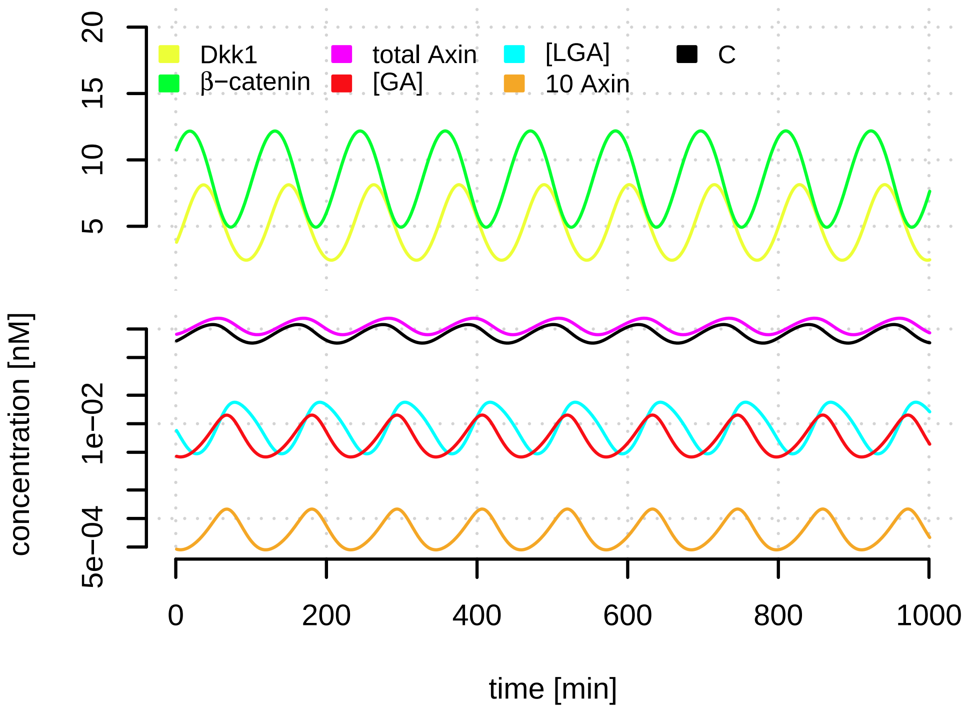

The Wnt model is simulated with a Hill coefficient of $h = 2$. The model still shows oscillations with a period of around 120 min, but the the affinity of the β-catenin to bind the $[GA]$ complex needs to be much higher than expected from experiments. The parameters used in this simulation ca be found in Table S1.
