# Supplementary material for: Dickkopf1 - A New Player in Modelling the Wnt Pathway
Source: PLoS One. 2011 Oct 12;6(10):e25550. doi: 10.1371/journal.pone.0025550 (PMC3192063; doi:10.1371/journal.pone.0025550)
Supplement: Figure S2 — Gaussian profiles of the Wnt gradient. (Top): Gaussian profiles of in the PSM are plotted with different initial values. A decreasing value of in the PSM will give rise to smaller amplitudes (middle) and slightly shorter/ almost constant periods (bottom). The abbreviation ref±i denotes the reference state value of ±i. (PDF) [file pone.0025550.s002.pdf]

Figure S2 - Gaussian profiles of the Wnt gradient

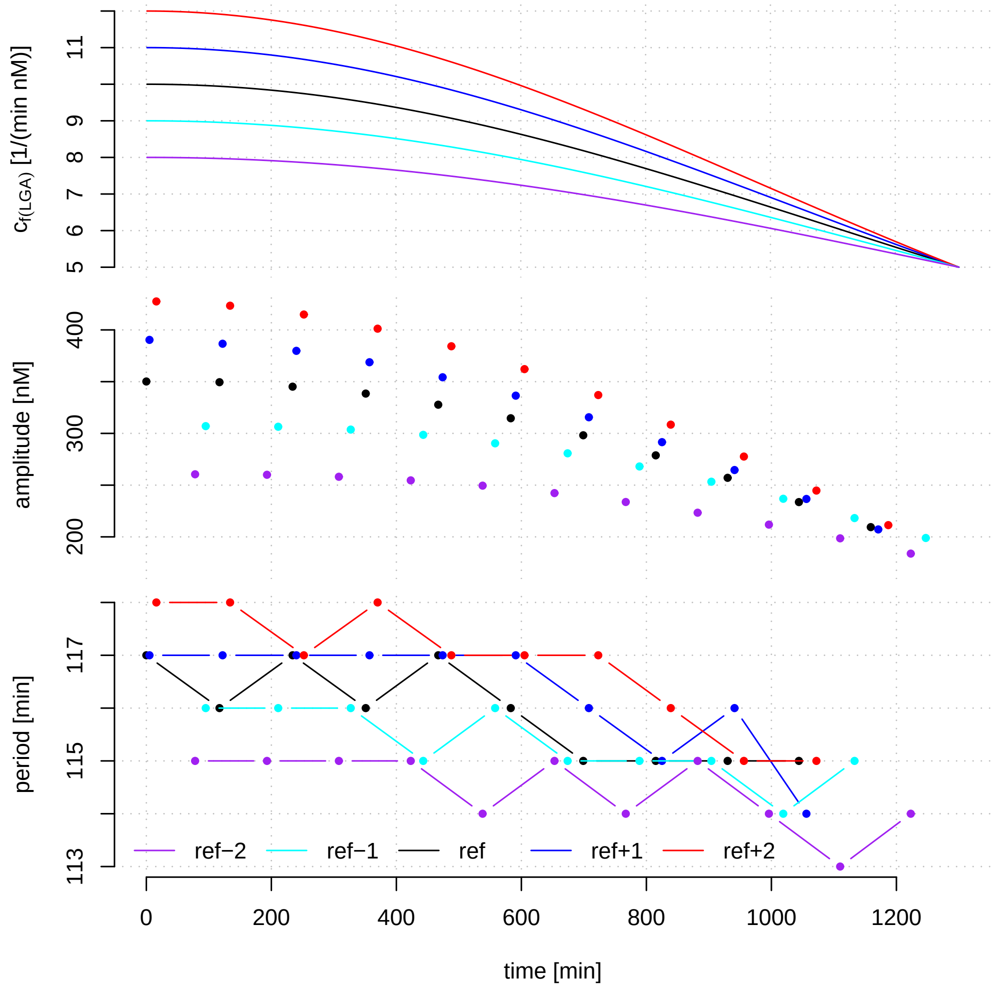

Top: Gaussian profiles of $c_{f[LGA]}$ in the PSM are plotted with different initial values. A decreasing value of $c_{f[LGA]}$ in the PSM will give rise to smaller amplitudes (**middle**) and slightly shorter/ almost constant periods (**bottom**). The abbreviation ref ± i denotes the reference state value of $c_{f[LGA]}$ $\pm i$.
