# Supplementary material for: Dickkopf1 - A New Player in Modelling the Wnt Pathway
Source: PLoS One. 2011 Oct 12;6(10):e25550. doi: 10.1371/journal.pone.0025550 (PMC3192063; doi:10.1371/journal.pone.0025550)
Supplement: Figure S3 — Desynchronization of neighboring cells. If the desynchronization between neighboring cells is small (A) then the oscillations of the Dkk1 level at the determination front is almost unaltered. However, if the desynchronization is strong then these oscillations are not appearing. (PDF) [file pone.0025550.s003.pdf]

Figure S3 - Desynchronization of neighboring cells

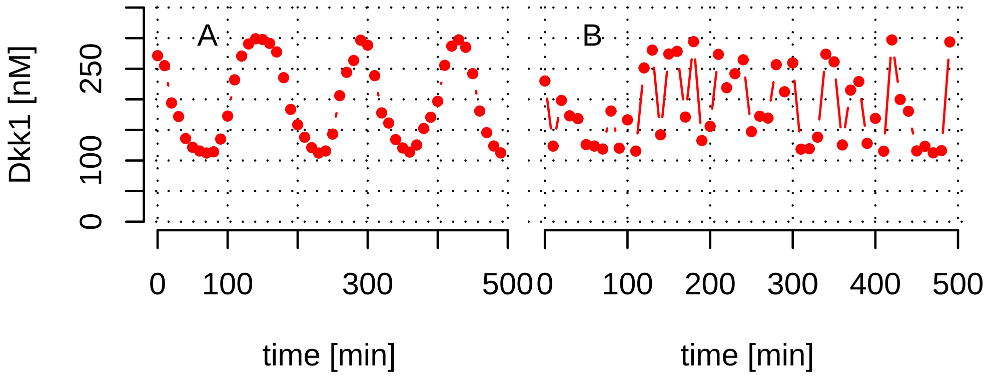

If the desynchronization between neighboring cells is small (**A**) then the oscillations of the Dkk1 level at the determination front is almost unaltered. However, if the desynchronization is strong then these oscillations are not appearing.
