# Supplementary material for: Dickkopf1 - A New Player in Modelling the Wnt Pathway
Source: PLoS One. 2011 Oct 12;6(10):e25550. doi: 10.1371/journal.pone.0025550 (PMC3192063; doi:10.1371/journal.pone.0025550)
Supplement: Figure S4 — The Wnt level in the tail bud. (A): When the Wnt level decreases in the tail bud, then the period of the Dkk1 level at the determination front extends. (B): The period is also extended when the Wnt level increases in the tail bud, but the periods drop significantly below the period in the reference state. (PDF) [file pone.0025550.s004.pdf]

Figure S4 - The Wnt level in the tail bud

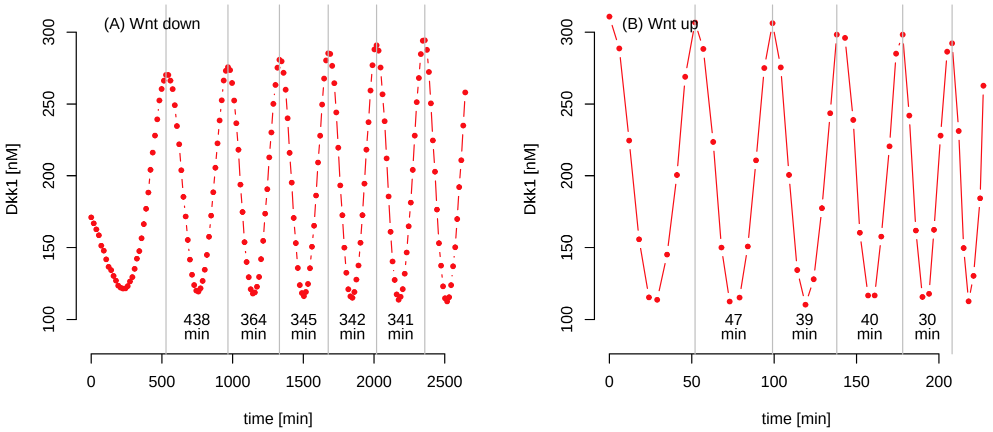

(**A**) When the Wnt level decreases in the tail bud, then the period of the Dkk1 level at the determination front extends. (**B**) The period is also extended when the Wnt level increases in the tail bud, but the periods drop significantly below the period in the reference state.
