# Supplementary material for: Dickkopf1 - A New Player in Modelling the Wnt Pathway
Source: PLoS One. 2011 Oct 12;6(10):e25550. doi: 10.1371/journal.pone.0025550 (PMC3192063; doi:10.1371/journal.pone.0025550)
Supplement: Table S1 — (PDF) [file pone.0025550.s005.pdf]

Table S1

Parameters in our model of the Wnt system with $h = 2$

Parameter	Process	Default Value
K_C	Dissociation constant C	$0.4\,\mathrm{nM}$
c_{bC}	Breaking of C	$16\,\mathrm{min}^{-1}$
α	Degradation of β-catenin	$10\,\mathrm{min}^{-1}$
$K_{[GA]}$	Dissociation constant $[GA]$	$0.5\,\mathrm{nM}$
$c_{b[GA]}$	Breaking of $[GA]$	$10\,\mathrm{min}^{-1}$
$K_{[LGA]}$	Dissociation constant $[LGA]$	$0.5\,\mathrm{nM}$
$c_{b[LGA]}$	Breaking of $[LGA]$	$10\,\mathrm{min}^{-1}$
ν	Degradation of Axin	$0.2\,\mathrm{min}^{-1}$
$K_{[LD]}$	Dissociation constant $[LD]$	$0.5\,\mathrm{nM}$
$c_{b[LD]}$	Breaking of $[LD]$	$0.02\,\mathrm{min}^{-1}$
S_B	Constant source of β-catenin	$0.9\,\mathrm{nM/min}$
S_A	Constant source of Axin	$0.002\,\mathrm{nM/min}$
c_{tsl}	Transcription of *dkk1*	$0.025\,\mathrm{min}^{-1}$
c_{tsc}	Translation of Dkk1 mRNA	$0.025\,(\mathrm{Nm}^2\,\mathrm{min})^{-1}$
τ_{Dm}	Average lifetime of dkk1 mRNA	$10\,\mathrm{min}$
τ_D	Average lifetime of Dkk1	$10\,\mathrm{min}$
$GSK3\beta_{tot}$	Total G level	$50\,\mathrm{nM}$
L_{tot}	Total L level	$7\,\mathrm{nM}$
